# EADC: An Efficient Anonymous Data Collection Scheme with Blockchain in Internet of Things

**DOI:** 10.3390/s24227162

**Published:** 2024-11-07

**Authors:** Zhiwei Si, Juhao Wang, Pengbiao Zhao, Xiaopei Wang, Jingcheng Song

**Affiliations:** 1 The School of Information Science and Technology, Linyi University, Linyi 276000, China; 220854042027@lyu.edu.cn (Z.S.); tsingtaowangjh@gmail.com (J.W.); 2 School of Information and Software Engineering, University of Electronic Science and Technology of China, Chengdu 610054, China; pengbiaozhao@std.uestc.edu.cn; 3 Department of Computer Science and Engineering, University of California, Riverside, CA 92521, USA; xwang605@ucr.edu

**Keywords:** anonymous data collection, privacy-preserving, blockchain, smart contracts, homomorphic encryption

## Abstract

The integration of smart contracts (SCs) within blockchain technology represents a pivotal direction in the evolution of the Internet of Things (IoT), enabling decentralization and enhancing user trust in the system. However, ensuring data privacy is a fundamental challenge that must be addressed during the deployment of these SCs. Many scholars have adopted data aggregation to protect privacy, but these methods are difficult to achieve fine-grained data collection. To this end, this paper proposes an efficient anonymous data collection (EADC) scheme suitable for the IoT environment. The scheme combines matrix algorithms with homomorphic encryption (HE) technology to effectively cut off the connection between users and data during data upload. In addition, the EADC scheme introduces a sophisticated data grouping protocol to improve the overall efficiency of the system. Analysis shows that the scheme can achieve efficient data collection without compromising user privacy.

## 1. Introduction

The origin of the Internet of Things (IoT) can be traced back to the 1980s. At that time, a Coke vending machine at Carnegie Mellon University in the United States was able to report inventory and temperature, becoming the first connected device [1]. In 1999, Professor Ashton proposed the concept of the “Internet of Things” [2,3]. After decades of development, the Internet of Things has become an important basic technology in the fields of smart grids, smart cities, smart medical care, internet of vehicles, and smart transportation [4]. With the increasing maturity of Internet of Things technology, smart sensors (SSs) such as smartphones, monitors, smart homes, and locators are widely deployed. These sensors collect and share massive amounts of data through the Internet [5]. Therefore, systems and applications can provide more accurate and timely feedback [6]. However, it relies on timely and accurate user data, which raises two pivotal issues: (1) users may distrust the system’s server, either withholding or falsifying data [7]; (2) users might believe their data will not be used appropriately, leading to similar data manipulation behaviors [8].

Blockchain technology is designed for decentralization, thereby bolstering user trust in the system [9]. It operates as a decentralized and unalterable ledger, recording transactions across a network of computers without a central authority. The essence of blockchain lies in its key features: decentralization ensures no single point of control, as data are managed by a network of nodes; transparency is guaranteed by public transaction records, allowing for verifiable history; and immutability makes data modification virtually impossible without network consensus [10]. These attributes inherently increase user trust in the system’s security and integrity. For the above problem (1), blockchain technology can already solve it well [11].

Smart contracts (SCs) are used for data collection in blockchain [12,13]. However, users’ data, in IoT, often involve sensitive information, which greatly reduces users’ willingness to share, as suggested by question (2) [14,15]. Therefore, how to collect user sensitive data while protecting data privacy has become a key issue facing the Internet of Things [16,17]. Privacy-preserving data aggregation technology is the main method to protect user sensitive information [18,19,20]. This technology encrypts the user’s sensitive data and uploads them to the aggregator or control center (CC) through homomorphic encryption (HE), masking, and differential privacy [21]. After decryption, the sum or average of multiple users’ data can be obtained. However, the aggregator or CC cannot obtain the plaintext data of a single user, thus effectively protecting user privacy [22]. This method performs particularly well in smart grids, air quality monitoring, and smart agriculture, which not only improves data collection efficiency but also protects user privacy [23,24,25].

Although privacy-preserving data aggregation can balance the mean and privacy issues of data collection in the above-mentioned fields, it has limitations in terms of fine-grained data collection requirements [26]. For example, in smart healthcare, if you want to obtain the fever level and incidence rate of urban residents after influenza, privacy-preserving data aggregation can only provide the sum or mean of these data but cannot reflect the detailed body temperature and specific number of people [27]. Similarly, in smart transportation, the precise location information of vehicles cannot be used to obtain useful detailed information through data aggregation. Additionally, smart meters collect users’ electricity consumption data through anonymization techniques, ensuring that user identities remain protected. Air quality monitoring networks anonymize pollutant data to prevent them from pointing to specific locations or individuals. Smart home systems analyze device usage patterns, leveraging anonymous data to optimize energy management without recording specific user information. These examples demonstrate the potential for effective data analysis while protecting privacy. Therefore, in these scenarios, privacy-preserving data aggregation is difficult to meet the needs of privacy protection and data availability at the same time [28,29].

Anonymous data collection technology can protect user privacy while collecting data in a fine-grained manner [30,31,32]. Specifically, anonymous data collection hides the user’s personal information in the mass information, cutting off the direct connection between the user and his or her data [33,34]. This allows the aggregator or CC to collect each user’s data, but it is impossible to associate these data with a specific user. For example, when obtaining the temperature status of residents in a city, the center for disease control and prevention (CDCP) can obtain the random arrangement of each user’s temperature through the mini-program, but it cannot locate the user based on the temperature information. This method can accurately understand the situation of abnormal body temperature in the city while protecting the privacy of residents. Therefore, even residents with a fever are willing to upload their real body temperature to the CDCP. In these scenarios, the collection of anonymized user data involves two main steps: privacy location assignment and anonymous data collection. Privacy location assignment allocates a unique slot to each user within the system, making it inaccessible to other users, thereby enhancing the privacy protection of the data collected. Privacy location allocation allocates a unique slot for each user in the system. This slot cannot be obtained by other users except the user himself. Anonymous data collection means to write local data into the allocated slot and fill other slots with 0. Then, this filled data string is encrypted and uploaded to the aggregator or CC. After decryption, the aggregator or CC can obtain the data of all users in the system. At present, although some schemes can achieve fine-grained data collection, they still face the following problems [17]: (1) Linear communication between users leads to large waiting delays. (2) The degree of trust assumption is high, and the assistance of a trusted third party is required. (3) The efficiency of anonymous data collection is low. (4) It is difficult to achieve multi-round location allocation.

In order to solve the above problems, an efficient anonymous data collection (EADC) scheme is proposed. EADC combines matrix eigenvalue and mask encryption technology to realize the parallel calculation of privacy location allocation between users. At the same time, this paper proposes a dual-key HE scheme to improve the efficiency of anonymous data collection. The main contributions of this paper include the following three aspects:

- A parallel computing anonymous data collection scheme is proposed, which reduces a lot of waiting delays compared to the previous linear communication method and greatly improves the communication efficiency of the system.
- Combined with privacy location allocation, the collection of original data is realized, and fine-grained access to user data is achieved. These data can reflect users’ behaviors and preferences more comprehensively and accurately, thus providing a better data basis for subsequent data analysis and applications.
- Through privacy location allocation combined with ElGamal encryption, the privacy of user behavior data is maintained. This technology combination combines two privacy protection methods to achieve double protection of user behavior data.
- A user grouping mode is proposed to improve the execution efficiency of system data collection, which means that the system can process large amounts of user data more efficiently and reduce computational complexity during the processing. By grouping users into categories or groups, the system can collect data for groups, resulting in an optimal allocation of resources.

The remainder of this manuscript is organized as follows. Section 2 is a preliminary section. Then, the model and design goal are described in Section 3. Section 4 presents the proposed scheme. Section 5 presents an efficiency and safety analysis. Finally, the paper is concluded in Section 6.

## 2. Preliminaries

### 2.1. User Grouping

CC generates collection group labels $\left\{T_{1},T_{2},\ldots ,T_{k}\right\}$ and sends them to all users. The user accepts the list $\left\{T_{1},T_{2},\ldots ,T_{k}\right\}$, selects a logo, and uploads his ID and label to CC. CC receives the labels and integrates it into multiple data collection groups based on the group labels uploaded by the user $\left\{M_{r}=\left\{ID_{i}|User_{i}\;selects\;T_{r}\right\}\right\},\left(r=1,2,\ldots ,k,i=1,2,\ldots ,n\right)$. CC saves and broadcasts the data collection groups to other entities.

### 2.2. Homomorphic Encryption

The public key *Y*, random number $\left\{r_{1},r_{2}\right\}$, and encryption algorithm Enc() are used to encrypt the plaintext data $\left\{m_{1},m_{2}\right\}$ according to Formulas (1) and (2), and the resulting ciphertext $\left(C_{1}^{a},C_{1}^{b}\right)$ and $\left(C_{2}^{a},C_{2}^{b}\right)$, where *G* is a generator. The aggregated ciphertext $Enc(m_{1}+m_{2})$ can be calculated by Equation (3), where $r_{3}=r_{1}+r_{2}$.

$$\begin{array}{ccc} Enc(m_{1}) & =(C_{1}^{a},C_{1}^{b}) & \left(1\right)\\ & =(r_{1}\cdot G,m_{1}\cdot G+r_{1}\cdot Y)\\ Enc(m_{2}) & =(C_{2}^{a},C_{2}^{b}) & \left(2\right)\\ & =(r_{2}\cdot G,m_{2}\cdot G+r_{2}\cdot Y)\\ Enc(m_{1}+m_{2}) & =Enc\left(m_{1}\right)+Enc\left(m_{2}\right)\\ & =(C_{1}^{a}+C_{2}^{a},C_{1}^{b}+C_{2}^{b}) & \left(3\right)\\ & =(r_{3}\cdot G,\left(m_{1}+m_{2}\right)\cdot G+r_{3}\cdot Y) \end{array}$$

### 2.3. Authenticated Encryption

Authenticated encryption ensures the confidentiality and integrity of information exchange between the sender and the receiver. The sender encrypts the plaintext data *m* into ciphertext *c* using the key *k*. The receiver decrypts the ciphertext using the same key to obtain the plaintext data. Authenticated encryption can be implemented using symmetric encryption, such as AES. Assuming that the key owned by both parties is set to *k*, the encryption process is written as AE.enc and the decryption process is written as AE.dec.

**Correctness** can be expressed as follows:

∀mand∀x

and we can obtain

AE.dec(k,c=AE.enc(k,m))=m

**Confidentiality** requires that for any $\left(m_{1},m_{2}\right)$, the ciphertext $\left(c_{1},c_{2}\right)$ obtained by executing the algorithm AE.enc with key *k* which cannot be distinguished by any adversary.

### 2.4. Privacy Location Assignment

Private location assignment uses cryptography and mathematical puzzles to anonymously rank users within a group to protect the user’s location information from being obtained by other users [35]. Users only know their own position in the ranking but not the positions of other users, thereby protecting user privacy. In this scheme, users upload their data to the corresponding location, encrypt them to form ciphertext and send the, to the recipient. After the receiver decrypts the aggregation, it obtains the original data of all members of the data collection group, but cannot map the data to specific users. This method effectively blocks the association between data and data sources and protects user privacy.

Because data are encrypted during transmission and processing, external malicious attackers cannot steal users’ personal data [36]. In addition, since users only know their own location and not the locations of other users, internal attacks are difficult to carry out, thus ensuring the security of the system.

### 2.5. Elliptic Curve Difficulty Problem

Elliptic curve cryptosystems are characterized by using shorter keys to achieve higher security and are therefore suitable for privacy protection in trustworthy systems. The elliptic curve equation defined as $y^{2}=x^{3}+a\cdot x+b\;mod\;p$ exists on the prime field $F_{p}$, where $a,b\in F_{p}$, *p* is a large prime number. The additive group of an elliptic curve *E* is of order *q*, where *G* is the generator.

- Elliptic Curve Discrete Logarithm Problem (ECDLP) assumption:
  Randomly pick two points $X=x\cdot G,x\in Z_{q}^{*}$ on the elliptic curve. Assume that *x* is obtained through *X* and *G*. The ECDLP assumption means that the probability of deducing *x* in probability polynomial time is ignored.
- Elliptic Curve Computational Diffie–Hellman Problem (ECCDHP) assumption:
  Randomly pick two points on the elliptic curve $X=x\cdot G,Y=y\cdot G,x,y\in Z_{q}^{*}$. Assume that x,y is obtained through x·y·G,G,X,Y. ECCDHP assumes that the computational advantage of completing the above challenge in polynomial time is negligible.

## 3. Models and Design Goal

This section describes the system model, attack model and design goals of the proposed scheme of EADC.

### 3.1. System Model

**Figure 1:**
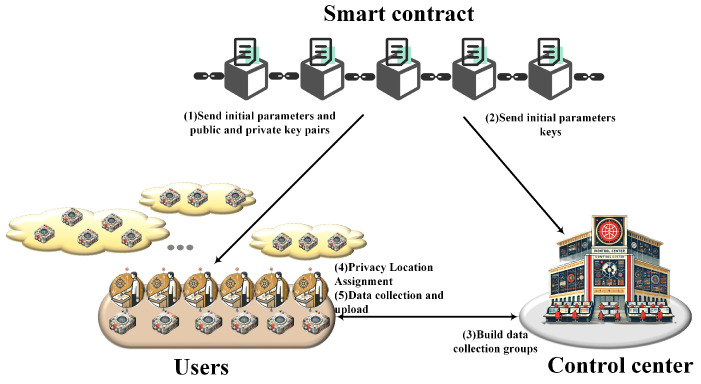
The system model for EADC.

In order to achieve a security trust assessment of SC, a complete IoT system requires a large amount of data collection, so a reliable communication model can provide security guarantees for it. As shown in Figure 1, EADC envisions efficiently transmitting data detected by users’ SSs to CC with the assistance of an SC, while protecting privacy and coping with complex network environments. In response to the above challenges, we assume that there are a large number of users in the model who can spontaneously form data collection groups. EADC decomposes the traditional overall data aggregation calculation into a small group calculation and re-aggregation mode, thereby achieving high efficiency of data processing and reducing the computational burden of SS and CC. The system model of the proposed solution consists of the following three entities:

Smart contract: The SC is responsible for distributing keys, encryption parameters, and similar assets to users and CC. Through SC, access to keys and system parameters is restricted to specific entities, ensuring data security and immutability.

Control center: CC has sufficient computing resources and is responsible for collecting data collected by the SS end and conducting analysis and feedback. Additionally, CC can communicate bi-directionally with users.

Users: Users are the owners of SSs and complete data collection together with CC. In addition, as uploaders of data within the system, users are very concerned about data security and privacy. Users can spontaneously form data collection groups as needed. For example, users with higher privacy requirements may choose groups with many members, while other users may choose groups with fewer members to increase the efficiency of data collection.

### 3.2. Attack Model

In the attacker model, we assume that users and CC are honest but curious. Specifically, users and CC follow protocols to complete their work, but obtain as much raw data from other users as possible. In addition, a SC is completely trustworthy, and any adversary attack is ineffective.

- Users and CC may use the information they have to try to decipher other users’ raw data.
- Although users pursue high trust scores, the EADC assumes that users are honest. Users may upload false data to disguise themselves, but this is beyond the scope of this article.
- External adversaries may eavesdrop on the data ciphertext sent by users in the public channel, but the original data are still safe.
- Adversary A may compromise the CC, but will not pose a threat to user data.

### 3.3. Design Goals

High execution efficiency: Compared with traditional data collection solutions, the EADC proposed in this article divides the original centralized full-member data aggregation calculation into multiple data collection groups and adopts a multi-block data collection calculation model. The smaller the number of users in the data collection group, the lower the computational complexity, which greatly improves the efficiency of data collection.

Privacy protection: In the data collection work, most solutions do not pay attention to the issue of protecting user privacy. However, user behavior data usually contain the user’s recent activities, which is the user’s sensitive information. Users do not want their private data to be obtained by others, which leads to their reluctance to share their data and may even lead to the problem of “data silos”. Therefore, this article adopts an anonymous collection method. By cutting off the connection between the user and the data source, other users or entities cannot determine the source of the data after obtaining the data, thus ultimately achieving the goal of protecting user privacy information. This approach addresses the need for user data privacy protection and facilitates breaking down barriers to information collection.

Original data collection: In privacy-preserving protocols, data aggregation is a common approach to protect private data which uses homomorphic or mask encryption to calculate the sum or average of user data in the system. However, CC cannot achieve fine-grained access to data. This article hopes that by combining anonymous collection methods, CC can obtain the user’s original data, but cannot match the collected data with the user, thereby achieving fine-grained collection of data while protecting user privacy.

## 4. Proposed Scheme: EADC

This section introduces an efficient group data collection scheme in IoT. EADC can effectively realize free grouping of users, allocation of private locations, and fine-grained collection of anonymized data. The solution is divided into four stages: parameter setup, user grouping, privacy location assignment, and data anonymization upload. The scheme operates between n users, a SC, and a CC. Table 1 lists the relevant symbols used in the article.

- Parameter setup: SC generates initial parameters within the system and sends them to the user through a secure channel, generates a public and private key pair for the user, calculates the parameters required for user encryption, and generates a decryption matrix for CC.
- User grouping: CC generates data collection group labels, users select and join the data collection group according to their needs, and then, CC is responsible for integrating these data collection groups.
- Privacy position allocation: At this stage, users in each data collection group are sorted for privacy. Each user can only obtain a unique position within the group, which prepares for subsequent anonymized data collection. Notice: For instance, in constructing matrix $A_{i}$ in Case 3, $A_{i}\left[i,i\right]$ denotes the element located at the intersection of the i-th row and i-th column on the diagonal of $User_{i}$’s matrix $A_{i}$. Additionally, $A_{i}\left[m,m\right]$ denotes the other diagonal elements of $User_{i}$’s matrix $A_{i}$, excluding $A_{i}\left[m,m\right]$. Details of how users perform privacy location encryption are shown in Algorithm 1, and details of how CC performs location decryption and obfuscation are shown in Algorithm 2.
- Anonymized data upload: According to the above results of privacy location allocation, users store data in locations within their respective groups, and other locations are filled with 0. Subsequently, the data are encrypted and uploaded using the homomorphism of the ElGamal cryptosystem, and finally decrypted by CC to obtain fine-grained anonymous data. The details of the user performing group data encryption are shown in Algorithm 3, and the details of CC performing aggregate decryption are shown in Algorithm 4.

**Figure 2:**
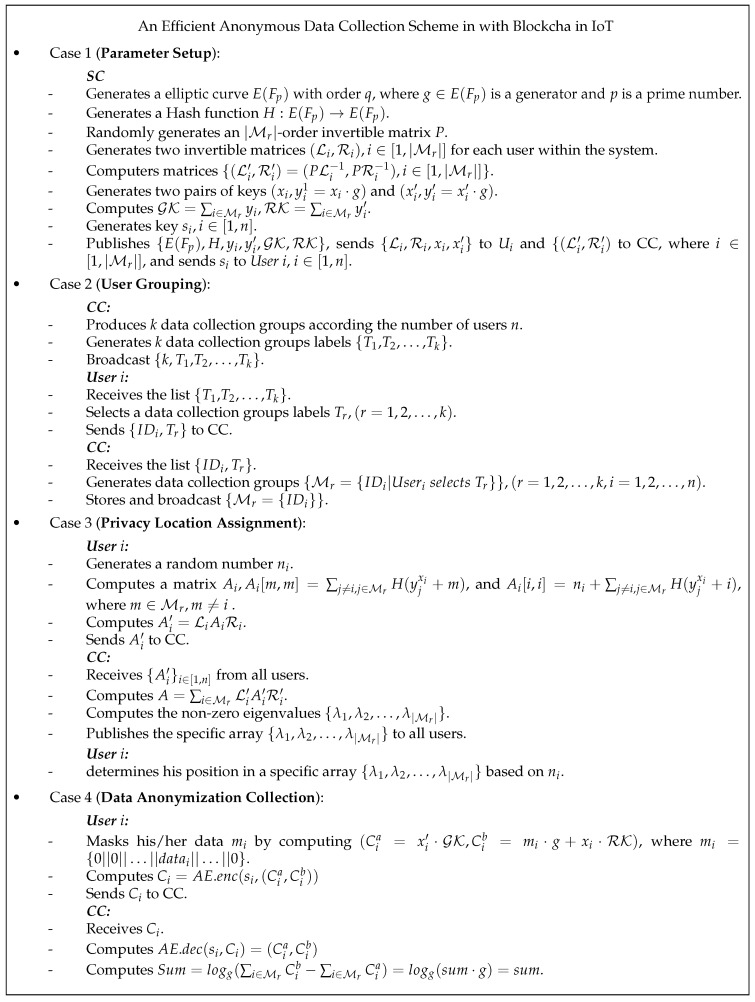
Detailed introduction of EADC.

Additional detailed information is provided in Figure 2.

Note: For convenience, only one data collection group is described in detail in Figure 2, and the model of other data collection groups is the same.
**Algorithm 1** Privacy location encryption at SS: **procedure**: **Input:** random number $n_{i}$, private key $x_{i}$, public key $y_{i}$, user serial number *i*, invertible matrices $\left(L_{i},R_{i}\right)$;: **Output:** secret location matrix Ai′;: **if** $j\in M_{r}\;and\;j\neq i$, then:    computes $A_{i}\left[m,m\right]=\sum_{j\neq i,j\in M_{r}}H\left(y_{j}^{x_{i}}+m\right)$;: **else**:    computes $A_{i}\left[i,i\right]=n_{i}+\sum_{j\neq i,j\in M_{r}}H\left(y_{j}^{x_{i}}+i\right)$;: **end if**;:    computes $A_{i}^{\prime }=L_{i}A_{i}R_{i}$;: **return** $A_{i}^{\prime }$;: **end procedure**

**Algorithm 2** Location decryption at CC
: **procedure**
: **Input:** received location encryption matrix Ai′, invertible matrices (Li′,Ri′);
: **Output:** location serial number $\left\{\lambda_{1},\lambda_{2},\ldots ,\lambda_{|M_{r}|}\right\}$;
: **if** $i\in M_{r}$, then
:    $A=\sum_{i\in M_{r}}L_{i}^{\prime }A_{i}^{\prime }R_{i}^{\prime }$;
: **else**
:    reject;
: **end if**;
: **return** $\left\{\lambda_{1},\lambda_{2},\ldots ,\lambda_{|M_{r}|}\right\}$;
: **end procedure**


**Algorithm 3** Data encryption at SS
: **procedure**
: **Input:** private keys $\left(x_{i},x_{i}^{\prime }\right)$, encryption parameters (GK,RK), data $m_{i}$, location serial numbe $\lambda_{i}$, location sequence $\left\{\lambda_{1},\lambda_{2},\ldots ,\lambda_{|M_{r}|}\right\}$;
: **Output:** ciphertext $\left(C_{i}^{a},C_{i}^{b}\right)$;
: **If** $i\in M_{r}\;and\;\lambda_{i}\in \left\{\lambda_{1},\lambda_{2},\ldots ,\lambda_{|M_{r}|}\right\}$, then
:    computes $\left(C_{i}^{a}=x_{i}^{\prime }\cdot \mathrm{GK},C_{i}^{b}=m_{i}\cdot g+x_{i}\cdot \mathrm{RK}\right)$;
: **else**
:    reject;
: **end if**;
: **return** $\left(C_{i}^{a},C_{i}^{b}\right)$;
: **end procedure**


**Algorithm 4** Data decryption at CC
: **procedure**
: **Input:** ciphertext $\left(C_{i}^{a},C_{i}^{b}\right)$;
: textbfOutput: sum = $\left\{data_{1}||data_{2}||\ldots ||data_{|M_{r}|}\right\}$;
: **If** $i\in M_{r}\;and\;C_{i}^{a},C_{i}^{b}\in E\left(F_{p}\right)$, then
: computes $Sum=log_{g}\left(\sum_{i\in M_{r}}C_{i}^{b}-\sum_{i\in M_{r}}C_{i}^{a}\right)$;
: **else**
:    reject;
: **end if**;
: **return** $\left\{data_{1}||data_{2}||\ldots ||data_{|M_{r}|}\right\}$;
: **end procedure**


## 5. Efficiency and Safety Analysis

This section analyzes EADC, mainly comparing it in three aspects: execution efficiency, privacy protection and original data collection, and comparing it with related privacy schemes.

### 5.1. High Execution Efficiency

In this section, the practicality and effectiveness of the proposed scheme will be demonstrated through functional and efficiency analysis.

#### 5.1.1. Experimental Setup

The goal of this scheme is to efficiently implement anonymous location allocation and data collection while minimizing computational and communication overhead on smart devices. Simultaneously, it is ensured the collected data is effectively protected throughout the entire process. To achieve this, experiments were conducted in three areas: functional analysis, computational overhead, and communication overhead. Additionally, to better demonstrate the functionality and efficiency of the proposed scheme, we compared it in detail with several other protocols. Given that the overhead for smart devices in this scheme involves anonymous location allocation and data collection, we selected two anonymous data collection schemes and two data aggregation schemes for comparison: Yao et al. [37], Wang et al. [38], Chen et al. [30], Zhu et al. [39] and Zhang et al. [40].

The experimental environment of this scheme is configured as follows: On a laptop equipped with Windows system (Wind 110, 64-bit), Intel Core i5-8300H CPU @ 2.30 GHz and 16.00 GB memory, a virtual machine with 1 CPU core and 2.0 GB RAM is configured, and the Ubuntu 18.04.1 LTS operating system is deployed on the virtual machine. The experimental code is developed in Python language and implemented in combination with the Charm-Crypto-0.50 framework. EADC uses Hyperledger Fabric as the development platform, and the version number is Hyperledger Fabric v1.4.3.

#### 5.1.2. Functional Comparison

The proposed scheme addresses the issue of requiring linear communication between users during anonymous location allocation while ensuring high efficiency and resistance to collusion attacks during data uploads. Additionally, compared to Zhang et al. [26] and other schemes, proposed scheme shifts the trust assumption from a fully trusted third party to an offline trusted third party. Because of the trusted third party, only one location allocation can be performed per initialization, limiting reusability and increasing data collection overhead. In addition, compared with Wang et al. [38], Chen et al. [30] schemes, parallel execution of private location allocation is achieved, which improves system efficiency. Notably, the proposed scheme includes a grouping protocol that allows data collection groups to be formed based on the preferences of users and servers, ensuring flexible data collection. For a clearer comparison of the security and practicality of the proposed scheme with other schemes, refer to Table 2 for details.

#### 5.1.3. Computational Overhead

Given the server’s superior computing power compared to the SS side, this paper focuses on the computational overhead on the SS side. This section simulates the SS side’s computational workload and compares the results with the Yao et al. [37], Wang et al. [38], Chen et al. [30], Zhu et al. [39] and Zhang et al. [40] schemes. For simplicity, we use symbols to represent the execution time and data length of the encryption operations. The execution time and data length of the encryption operations are shown in Table 3. Notably, the elliptic curve operation is performed on the *MNT224* elliptic curve.

Privacy location assignment phase: In the proposed scheme, participants generate a secret matrix and fill its parameters using masks. The SS’s computational complexity during the filling process is $O(n^{2})$, representing the primary overhead at this stage. To protect the privacy of the secret matrix, the scheme employs two confusion matrices to encrypt it. The computational complexity of addition, exponentiation, hashing, and matrix multiplication is $O(n^{2})$, O(n), $O(n^{2})$, and O(1), respectively.

In contrast, the Yao et al. [37], Wang et al. [38] and Chen et al. [30] schemes use the Shuffle mechanism for privacy location assignment, where users engage in linear communication. The computational complexity of multiplication, exponentiation, and inversion for each user is O(n). The proposed scheme, however, utilizes parallel communication for anonymous location allocation, whereas the Yao et al. [37], Wang et al. [38] and Chen et al. [30] schemes depend on linear communication between users. As a result, the computational complexity for overall location allocation in the proposed scheme is $O(n^{2})$ on the user side, compared to n*O(n) for the Yao et al. [37], Wang et al. [38] and Chen et al. [30] schemes.

**Figure 3:**
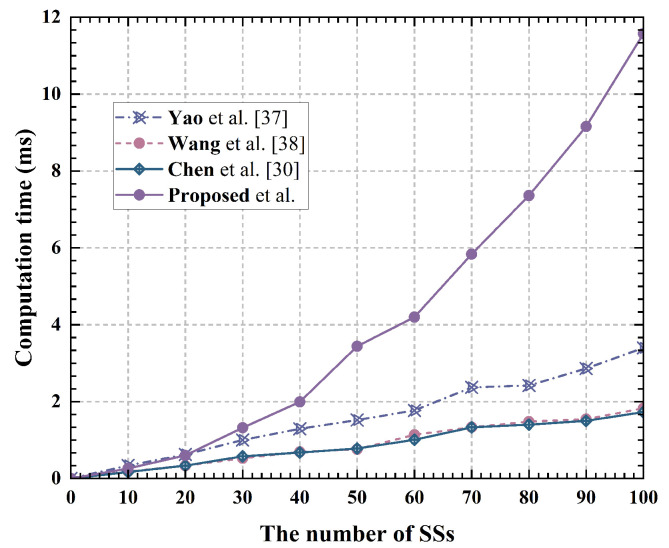
Computational time of a single user at SS [30,37,38].

**Figure 4:**
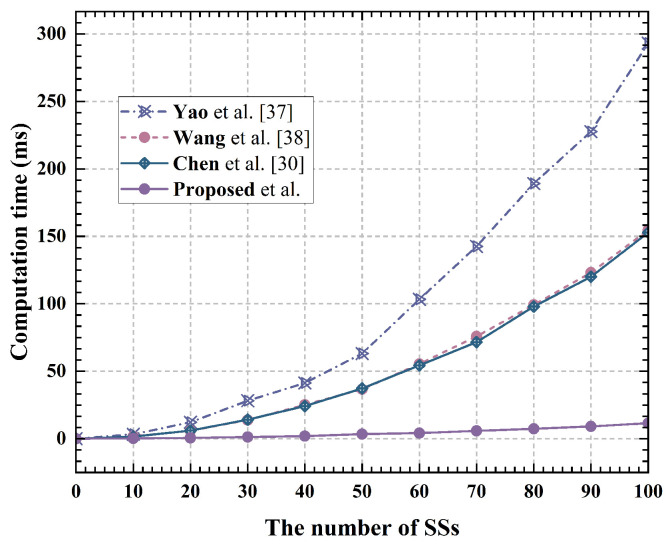
Computational time of completing protocol at SS [30,37,38].

The computational time required for a single user to perform privacy location assignment with varying numbers of system users is illustrated in Figure 3. The total computational time required for the user side to complete privacy location assignment as the number of system users varies is depicted in Figure 4 As depicted in Figure 3, the proposed solution’s computational overhead for users is higher than that of the Shuffle mechanism. However, because the proposed solution employs parallel computing, it eliminates waiting delays $T_{w}$ between users and significantly enhances the efficiency of anonymous allocation, as shown in Figure 4.

**Figure 5:**
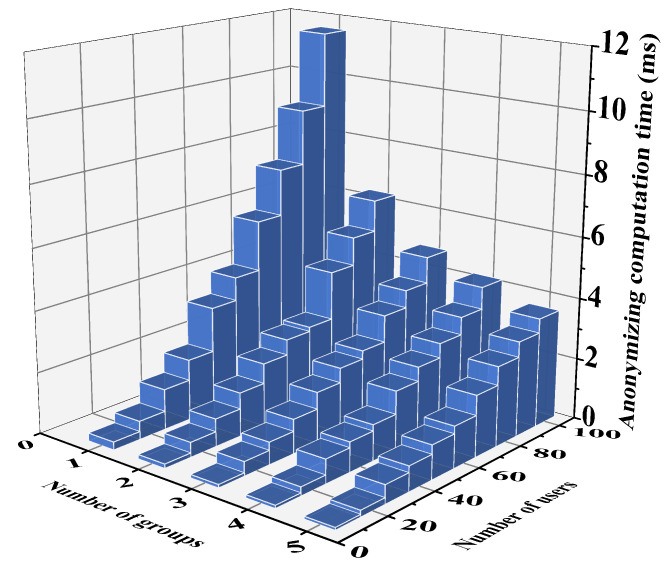
Computation time for grouping at SS.

It is important to note that in scenarios with a large number of users, the proposed anonymous collection protocol may impose significant computational burdens on both the client and server, particularly on the user side. To address this, the paper proposes a grouping scheme to reduce the computational overhead on the user side. The client’s computational complexity is $O(n^{2})$ during protocol execution, and the scheme divides the n users into *k* anonymous collection groups, which then execute the proposed scheme simultaneously. The grouping protocol effectively alleviates the computational complexity issues for the client in multi-user scenarios. The computational time of the proposed scheme after grouping, with different numbers of system users, is illustrated in Figure 5. The results indicate that the computational burden on the SS side is further reduced after grouping.

**Figure 6:**
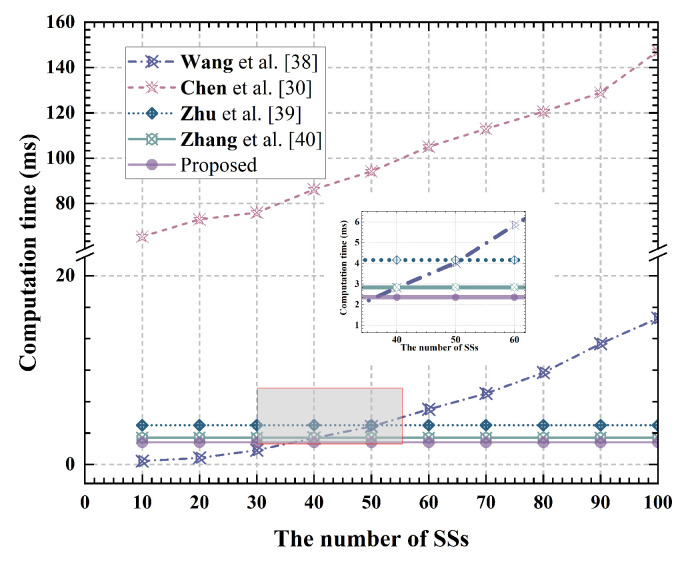
Computation time during the data collection phase at SS [30,38,39,40].

Data anonymization collection phase: In the data collection phase, the proposed scheme conceals the source data using two group keys and performs two elliptic curve addition and three elliptic curve multiplication operations. In contrast, the Wang et al. [38] scheme utilizes session keys and Hash functions to perform XOR operations to mask the original data, with a complexity of $O(n^{2})$ for Hash and XOR operations. The Chen et al. [30] scheme employs Shamir secret sharing to hide data within polynomial coefficients. While it offers high security, its computational overhead is substantial. The Zhu et al. [39] and Zhang et al. [40] schemes operate on elliptic curves with a complexity of O(1), making them relatively efficient. Figure 6 presents the computation time required for each scheme to perform data collection on the SS side, based on varying numbers of system users. The results indicate that when the number of system users is below 30, the Wang et al. scheme is the most efficient; however, when the user count exceeds 30, the proposed scheme demonstrates higher efficiency.

In summary, the detailed comparison of the computational cost of each solution is shown in Table 4.

#### 5.1.4. Communication Overhead

**Figure 7:**
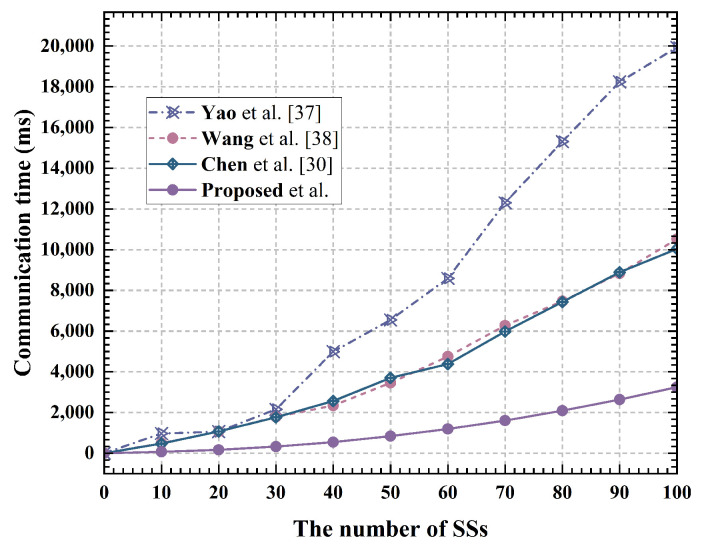
Communication time at SS [30,37,38].

**Figure 8:**
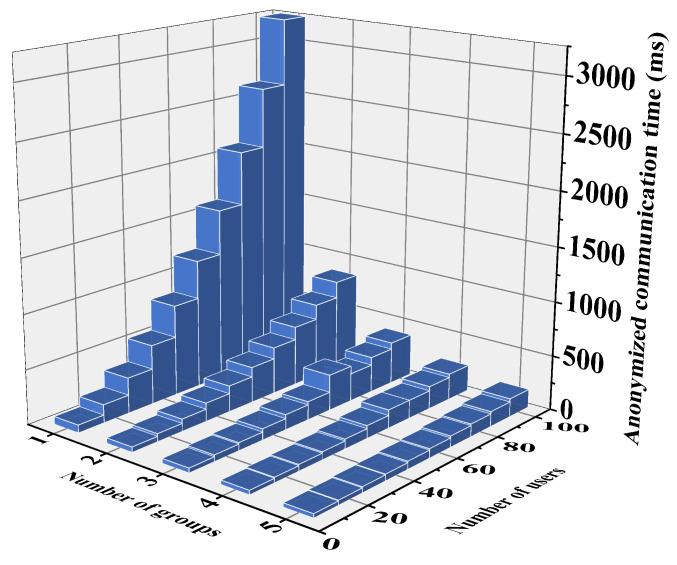
Communication time for grouping at SS.

The focus of this article is to complete data collection while ensuring privacy. No specific identity authentication scheme is added to the protocol. Therefore, the privacy location allocation process should be the focus of comparison, as follows:

Privacy location assignment phase: The proposed scheme uploads a matrix to the server, resulting in a space complexity of $O(n^{2})$. In contrast, the Yao et al. [37], Wang et al. [38] and Chen et al. [30] schemes upload ciphertext encrypted by multiple users, with a space complexity of O(n). Specifically, the proposed scheme requires a single SS to upload data of length $n^{2}L_{c_{i}}$ to the server, while in the Yao et al. [37], Wang et al. [38] and Chen et al. [38] schemes, each user uploads data of length $nL_{c_{i}}$. However, the proposed scheme uploads location ciphertext in parallel, whereas SS in the Shuffle scheme uploads ciphertext through linear communication. Therefore, on the SS side, the total transmission data length required by the proposed scheme for anonymous location allocation is $n^{2}L_{c_{i}}$, compared to $nL_{c_{i}}$ in the Yao et al. [37], Wang et al. [38] and Chen et al. [30] schemes. The specific time required for communication is illustrated in Figure 7. As shown in the figure, while the data length transmitted by the proposed scheme and the Yao et al. [37], Wang et al. [38] and Chen et al. [30] schemes at the SS side is the same, the transmission time varies significantly. This is due to the higher efficiency of transmitting $n^{2}L_{c_{i}}$ data length in one instance compared to transmitting $nL_{c_{i}}$ data length multiple times. To further enhance data transmission efficiency, a clear grouping scheme is proposed in the plan. Figure 8 presents the communication time of the proposed scheme after grouping, with varying numbers of system users. The results indicate a significant improvement in efficiency after grouping.

Data anonymization collection phase: Since this solution does not involve identity authentication, the length of data uploaded by each solution is the same under the same data length, so it will not be discussed in detail.

#### 5.1.5. Throughput of Smart Contracts

**Figure 9:**
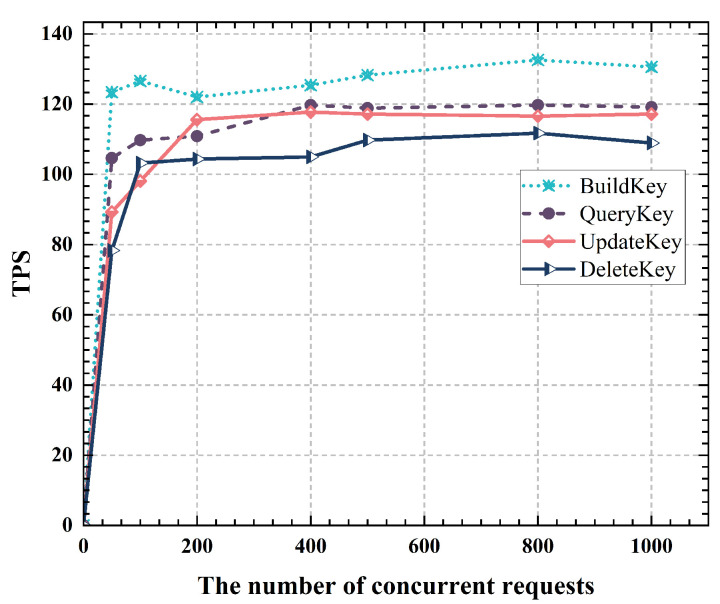
Throughput under different concurrent requests.

This section tests the impact of various SC operations in EADC on system throughput. First, the maximum transactions per block is set to 50. Based on this setting, concurrent transactions are tested at 100, 200, 400, 500, and 800 to evaluate the throughput performance of each SC operation. The experimental results are shown in Figure 9. BuildKey denotes the key generation operation in SC, QueryKey refers to the key query, UpdateKey indicates the key update, and DeletedKey signifies the key deletion operation.

According to the experimental results, throughput for key generation in SCs is the highest, with key query and update operations having similar throughput, and key deletion showing the lowest throughput. With an increasing number of concurrent transactions, the throughput of each SC operation rises gradually until it reaches stability. Stability occurs once the number of connections in the blockchain network’s connection pool hits its upper limit, causing system throughput to stop rising and remain constant.

### 5.2. Privacy Protection

The data sent by the user to CC are transmitted over an open channel, making it easy for external attackers to access these ciphertexts. However, because of AES and ElGamal homomorphic encryption, external adversaries are unable to access the key $s_{i}$ of user $User_{i}$, preventing them from analyzing the ciphertext $C_{i}$ [41,42].

In addition, if internal adversaries want to obtain the data information of other users, they can only be cracked by obtaining the private key $\left\{x_{i},x_{i}^{\prime }\right\}$, but obtaining the private key through the public keys $\left\{y_{i},y_{i}^{\prime }\right\}$ requires solving the Diffie–Hellman difficulty assumption. Therefore, users cannot obtain behavioral data information of other users.

### 5.3. Original Data Collection

Combining location allocation and privacy-preserving data collection methods, a system solution can be constructed that simultaneously protects user privacy and achieves fine-grained data collection. In this scheme, users’ raw data are anonymized before being collected and transmitted without being directly exposed to CC. Compared with traditional data collection solutions, this method can obtain data information of users, rather than in the form of group data sum, while cutting off the connection between users and data to ensure privacy.

Finally, the internal adversary CC can only obtain the total data of users within the system. Although these sums are behavioral data for a single user, CC does not know the owner of each data due to the use of anonymity methods. Therefore, CC cannot find the corresponding user based on the fine-grained data collected to protect user privacy.

## 6. Conclusions

This paper proposes an efficient group data collection scheme with blockchain in IoT. Blockchain is adopted for decentralization, thereby bolstering user trust for systems. Moreover, EADC adopts the grouping mode, which effectively reduces the data aggregation system with blockchain complexity. This solution combines privacy location allocation to achieve private collection of user behavior data. It essentially cuts off the connection between data and data sources, ensuring fine-grained access and privacy of user data. Simultaneously, it provides new ideas for privacy data collection for IoT. Finally, the performance and safety analysis results show that the design objectives are fully met.

## Figures and Tables

**Table 1:** Definition of symbols.

Acronym	Descriptions
$U_{i}$	User
$ID_{i}$	Users’ identity document
CC	CC is the Control Center
SC	Smart contract
H(x)	Hash function
$L_{i},R_{i}$	Nvertible matrices of $U_{i}$
P	Security matrix
$\lambda_{i}$	location serial number
xi,xi′	Private key of $U_{i}$
yi,yi′	Public key of $U_{i}$
$T_{i}$	Data collection groups labels
$M_{r}$	Data collection group
$m_{i}$	Behavioral data information of $U_{i}$
$C_{i}^{a},C_{i}^{b}$	Encrypted behavioral data information of $U_{i}$
Sum	Aggregated behavioral data information of $U_{i}$
RK,GK	Parameters used for encryption within the data collection group

**Table 2:** Function comparison with other schemes.

Protocol	Security	Privacy	Agility	Anti-Collusion Attack	No TTP	Location Allocation Reusability	Parallel Execution
Zhang et al. [26]	●	●	○	●	○	○	●
Zhao et al. [28]	●	●	○	●	●	○	●
Wang et al. [38]	●	●	○	○	●	○	○
Chen et al. [30]	●	●	○	●	●	●	○
Zhu et al. [39]	●	●	○	●	○	-	-
Zhang et al. [40]	●	●	○	●	○	-	-
EADC	●	●	●	●	●	●	●

The symbol (0,0) circle (0.7ex); indicates YES, (0,0) circle (0.7ex); indicates NO, - indicates no anonymous process.

**Table 3:** Cryptographic operation and execution time.

Notation	Description of Cryptographic Operations
$T_{ecc}^{ADD}$	The time to compute point addition operation based on elliptic curve cryptography
$T_{ecc}^{MUL}$	The time to perform scalar multiplication operation based on elliptic curve cryptography
$T_{Xor}$	The time of one XOR operation on cyclic group G
$T_{a}$	The time of one addition operation on cyclic group G
$T_{m}$	The time of one multiplication operation on cyclic group G
$T_{e}$	The time of one exponential operation on cyclic group G
$T_{s}$	The time to perform one Shamir secret sharing operation on cyclic group G
$T_{H}$	The time of one-way hash operation on cyclic group G
$T_{i}$	The time of one inversion operation on cyclic group G
$T_{tr}$	The time of one matrix multiplication operation on cyclic group G
$L_{c_{i}}$	The data length of one ciphertext
$T(L_{c_{i}})$	The time to transmit one ciphertext
$T_{w}$	Waiting delay

**Table 4:** Computation cost comparison.

Protocol	Privacy Location Allocation	Anonymous Data Collection	Computation Total Cost
Yao et al. [37]	$nT_{m}+3nT_{e}+nT_{i}$	-	$nT_{m}+3nT_{e}+nT_{i}$
Wang et al. [38]	$\left(n+2\right)T_{m}+\left(\left(3/2\right)n+2\right)T_{e}+\left(n/2\right)T_{i}$	$n^{2}T_{H}+\left(n^{2}-n+1\right)T_{Xor}$	$\left(n+2\right)T_{m}+\left(\left(3/2\right)n+2\right)T_{e}+\left(n/2\right)T_{i}+n^{2}T_{H}+\left(n^{2}-n+1\right)T_{Xor}$
Chen et al. [30]	$\left(n+2\right)T_{m}+\left(\left(3/2\right)n+2\right)T_{e}+\left(n/2\right)T_{i}$	$T_{s}+\left(n^{2}+n-1\right)T_{a}+n^{2}T_{h}$	$T_{s}+\left(n/2\right)T_{i}+\left(n^{2}+2\right)T_{m}+\left(\left(3/2\right)n+3\right)T_{e}+\left(n^{2}+n-1\right)T_{a}+n^{2}T_{H}$
Zhu et al. [39]	-	$2T_{a}+5T_{e}+3T_{ecc}^{ADD}+3T_{H}$	$2T_{a}+5T_{e}+3T_{ecc}^{ADD}+3T_{H}$
Zhang et al. [40]	-	$2T_{a}+3T_{ecc}^{ADD}+T_{e}$	$2T_{a}+3T_{ecc}^{ADD}+T_{e}$
EADC	$\left(n^{2}-1\right)T_{a}+\left(n-1\right)T_{e}+2T_{matr}+n^{2}T_{H}$	$3T_{ecc}^{MUL}+T_{ecc}^{ADD}$	$3T_{ecc}^{MUL}+T_{ecc}^{ADD}+\left(n^{2}-1\right)T_{a}+\left(n-1\right)T_{e}+2T_{matr}+n^{2}T_{H}$

## Data Availability

The original contributions presented in the study are included in the article, further inquiries can be directed to the corresponding author.
